# Roles of Phyllosphere Microbes in Rice Health and Productivity

**DOI:** 10.3390/plants13233268

**Published:** 2024-11-21

**Authors:** Andrews Danso Ofori, Wei Su, Tengda Zheng, Osmond Datsomor, John Kwame Titriku, Xing Xiang, Abdul Ghani Kandhro, Muhammad Irfan Ahmed, Edzesi Wisdom Mawuli, Richard Tuyee Awuah, Aiping Zheng

**Affiliations:** 1 State Key Laboratory of Crop Gene Exploration and Utilization in Southwest China, Sichuan Agricultural University, Chengdu 611130, China; andrewsdanso@icloud.com (A.D.O.);; 2 Department of Plant Pathology, Rice Research Institute, Sichuan Agricultural University, Chengdu 611130, China; 3 Renshou County Agricultural and Rural Bureau, Meishan 620500, China; 4 College of Animal Science and Technology, Yangzhou University, Yangzhou 225009, China; 5 College of Agronomy, Sichuan Agricultural University, Chengdu 611130, China; 6 Plant Improvement and Productivity Division, Biotechnology Unit, Council for Scientific and Industrial Research, Fumesua, Kumasi P.O. Box UP 63, Ghana; 7 Crop and Soil Science Department, Faculty of Agriculture, Kwame Nkrumah University of Science and Technology (KNUST), PMB KNUST, Kumasi P.O. Box UP 1279, Ghana

**Keywords:** phyllosphere, microbial communities, plant health, rice plant, plant–microbes interaction, microbial diversity

## Abstract

The phyllosphere, comprising the aerial portions of plants, is a vibrant ecosystem teeming with diverse microorganisms crucial for plant health and productivity. This review examines the functional roles of phyllosphere microorganisms in rice (*Oryza sativa*), focusing on their importance in nutrient uptake, disease resistance, and growth promotion. The molecular mechanisms underlying these interactions are explored along with their potential applications in enhancing sustainable rice production. The symbiotic relationships between rice plants and their associated microorganisms are highlighted, offering insights into improved agricultural practices. Furthermore, this review addresses the challenges and future developments in translating laboratory findings into practical applications. By synthesizing current research, this comprehensive analysis serves as a valuable resource for leveraging phyllosphere microbes in rice farming and related fields.

## 1. Introduction

The rice phyllosphere, representing the aerial parts of the plant, is a vast ecosystem hosting diverse microorganisms, including archaea, bacteria, fungi, protozoa, cyanobacteria, viruses, and nematodes [1]. This microbiota provides essential functions for plant health, such as nutrient acquisition, stress resilience, and growth support [2].

Due to cultivation challenges, researchers often use culture-independent strategies to study phyllosphere microbial communities [3]. These communities include plant pathogens, ice-nucleation-active bacteria, decomposers, phytohormone producers, nitrogen fixers, and pathogen antagonists [4]. The 16S rRNA gene amplicon sequencing approach has been effective for investigating the structure, arrangement, and temporal–spatial dynamics of microbial communities in the phyllosphere [4].

Rice (*Oryza sativa* L.) is a crucial global crop, feeding over half the world’s population [5,6] and providing more than 23% of global calorie intake. It is predominantly grown in Asia, particularly in South and Southeast Asian countries [7], Latin America, and Africa [8], significantly affecting many economies. Rice production is expected to increase by 40% by 2030 to meet the rising demand [9]. By enhancing the microbial communities in the rice phyllosphere, we can potentially improve crop resilience and yield, thereby addressing the challenges posed by increased production needs.

The phyllosphere provides a unique habitat for microbes despite extreme conditions. Although the phyllosphere hosts beneficial microbes, it can also harbor potential pathogens [10]. Understanding these complex interactions within the phyllosphere is crucial for developing innovative disease control strategies and promoting sustainable agriculture [11].

Advances in metagenomics and bioinformatics have provided unprecedented insights into the phyllosphere microbial composition [12]. However, a comprehensive understanding of how these communities function, interact, and influence rice plant health and productivity is still lacking, especially in the context of global challenges such as climate change [13].

This review aims to investigate the diversity and functional roles of phyllosphere microorganisms in rice health and productivity, elucidate the underlying molecular mechanisms, discuss potential applications for sustainable rice production, and address challenges in translating laboratory findings into practical agricultural applications. While bacteria dominate the current research, future studies could explore the roles of phyllosphere fungi and viruses in nutrient cycling and pathogen suppression, particularly using metagenomics to uncover latent symbiotic relationships.

## 2. Microbial Diversity in the Rice Phyllosphere

The rice phyllosphere is characterized by a rich and complex microbial community that plays a pivotal role in the ecological dynamics of rice plants. This section explores the composition, diversity, and functional roles of these microorganisms, highlighting their interactions with the host plant and their contributions to overall plant health and productivity

### 2.1. Bacterial Communities in the Rice Phyllosphere

The rice phyllosphere hosts a diverse array of bacteria, including *Methylobacterium*, *Pantoea*, *Sphingomonas*, *Pseudomonas*, and *Bacillus*, which are consistently found across different rice genotypes and environmental conditions and with each exhibiting distinct physiological adaptations for survival in this specialized environment [14,15]. These microorganisms play a crucial role in shaping microbial dynamics within the rice phyllosphere and can be categorized based on their relationship with plants as endophytic, epiphytic, pathogenic, or non-pathogenic [14]. Bacterial populations within the phyllosphere can reach densities ranging from 10^5^ to 10^7^ cells per cm^2^ of leaf area, with prominent phyla including *Proteobacteria*, *Actinobacteria*, and *Bacteroidetes* [16,17]. According to research in Malaysia and Bangladesh, specific phyllosphere bacteria such as *Pseudomonas fluorescens* and *Pseudomonas aeruginosa* exhibit promising potential as biocontrol agents for rice diseases like sheath blight. These isolates effectively inhibit fungal pathogens and promote plant growth, underscoring their utility in disease management and yield improvement [18].

*Methylobacterium*, which is prevalent in the phyllosphere of wild rice, can fix nitrogen and produce plant-growth-promoting compounds such as cytokinins, offering significant growth advantages to host plants [19,20]. Moreover, studies on *Methylobacterium* strains in northeastern India have shown varied effects on rice growth and yield. Although some strains have proven beneficial, others exhibit minimal impact, highlighting the importance of carefully selecting compatible host–bacterium pairs to optimize agricultural outcomes [19]. Additionally, bacteria such as *Lactobacillus* spp. and *Aspergillus* spp. contribute to disease resistance against rice false-smut disease by inducing apoptosis-like cell death via H_2_O_2_ overproduction [21]. Despite the diverse bacteria and their varying effects, the population dynamics of phyllosphere bacteria remain stable over time, balancing growth and death under fluctuating conditions [1].

**Figure 1:**
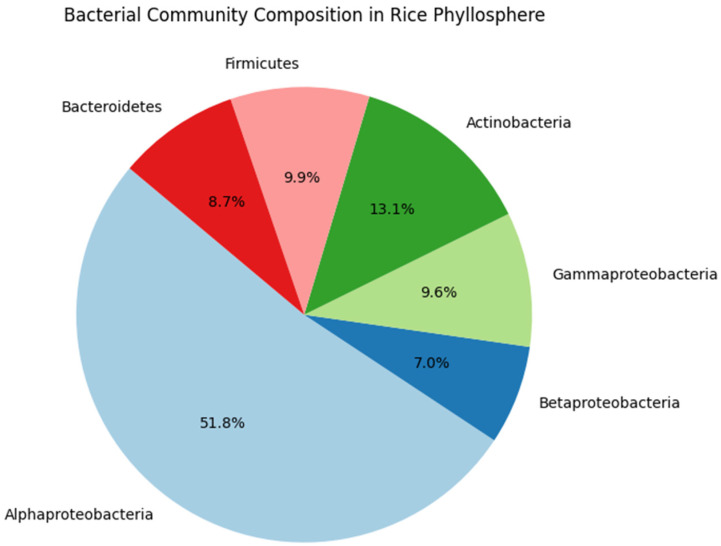
Relative abundance of bacterial genera in the rice phyllosphere. Sources: [17,44,45,46]. **Note:** Data for this figure were compiled from various studies on bacterial communities in rice phyllosphere, highlighting their relative abundances.

Santosa et al. [22] examined growth-promoting bacteria in the leaves of the rice cultivar IR-64 and found that their presence led to notable increases in plant height, upper part biomass, and root biomass. Investigations into phyllosphere bacteria for managing blast diseases have highlighted *Bacillus subtilis* subsp. *subtilis* as capable of synthesizing antifungal compounds and inducing systemic resistance [23,24]. This strain activates defense-associated enzymes such as peroxidase and polyphenol oxidase in rice foliage, suggesting its potential to activate the plant’s innate defensive mechanisms and enhance resilience against rice blast disease [25,26]. Although the efficacy of *Bacillus subtilis* in controlling *Magnaporthe oryzae* has been well documented in several studies, other studies have reported inconsistent results, likely due to varying environmental conditions. To address these discrepancies, future research should prioritize comparative field trials across diverse ecosystems to better understand the factors influencing their effectiveness [27]. Table 1 summarizes the functional roles of dominant bacterial taxa in the rice phyllosphere. Moreover, Figure 1 also illustrates the relative abundance of various bacterial genera in the rice phyllosphere, highlighting the dominance across different rice genotypes, with significant implications for plant health and disease resistance.

In addition to the diverse bacterial communities, the rice phyllosphere also hosts a variety of fungi that play crucial roles in plant health and productivity.

### 2.2. Fungi in the Rice Phyllosphere

The fungi inhabiting the rice phyllosphere can be broadly classified into epiphytic and endophytic groups. Epiphytic fungi reside on the surfaces of rice leaves, stems, and flowers and serve as the initial points of interaction with the external environment. Although bacteria are widely recognized for their influence on rice health, endophytic fungi, such as *Trichoderma* and *Fusarium*, have demonstrated significant biocontrol abilities, enhancing the resistance of plants to pathogens, such as *Rhizoctonia solani* and *Magnaporthe oryzae*. The rice phyllosphere hosts a diverse and abundant community of epiphytic fungi, including a variety of species like *Penicillium*, *Fusarium*, *Aspergillus*, and yeasts such as *Pseudozyma antarctica* and *Bullera japonica* [47,48]. They act as biocontrol agents, suppress pathogens, and act as pathogens themselves. Various fungal epiphytes have been observed to antagonize the rice blast fungus *Pyricularia oryzae* through mechanisms such as mycelial overgrowth, colonization, and antibiosis. Remarkably, *Penicillium* sp. GM15 and *Fusarium* sp. LM1 exhibits parasitic activity against *P. oryzae* mycelia, suggesting its promising role as a biocontrol agent [47]. Furthermore, epiphytic fungi, in combination with bacteria, have demonstrated effectiveness in suppressing the rice sheath blight caused by *Rhizoctonia solani*. Among them, *Aspergillus niger* has been recognized as a strong antagonist, significantly decreasing disease severity under field conditions [49].

In addition to epiphytic fungi, endophytic fungi colonizing the internal tissues of rice plants contribute significantly to the phyllosphere microbiome. These fungi often establish mutualistic relationships with the host plant, enhancing nutrient uptake, providing drought resistance, and offering protection against pathogens [50]. They enhance plant growth and development through the production of growth-promoting compounds such as gibberellic acid and ammonia, along with the solubilization of essential nutrients such as phosphate and zinc [51,52].

One study by Putri et al. [53] investigated the role of endophytic fungi in controlling *M. oryzae*, the pathogen responsible for rice blast diseases. They isolated several fungal endophytes from rice plants and discovered that certain strains of *Trichoderma* and *Fusarium* exhibit strong antagonistic activity against *M. oryzae*. The suppression of rice blast was due to both the direct inhibition of the pathogen and the induction of systemic resistance in the host plant, showing potential for using these endophytes as biocontrol agents instead of chemical fungicides [54]

In another study, Gao et al. [55] screened phyllosphere endophytic fungi for their ability to suppress Rhizoctonia solani, the pathogen responsible for rice sheath blight. They found that a strain of *Chaetomium spirale*, ND35, significantly reduced sheath blight severity by producing antifungal enzymes such as chitinases and glucanases and by inducing defense responses in rice plants. This emphasizes the potential of phyllosphere fungi as biocontrol agents against economically significant diseases in rice.

Deb et al. [56] investigated the antifungal properties of native *Beauveria bassiana* isolates against *R. solani*, the pathogen responsible for rice sheath blight. The study found that *B. bassiana* exhibited antagonistic activity through the production of enzymes such as chitinase and glucanase. A combined approach, using *B. bassiana* as both a seed treatment and foliar spray, was effective in reducing disease incidence and promoting plant growth under field conditions.

Advancements in molecular methodologies, notably internal transcribed spacer (ITS) sequencing, have significantly enhanced our understanding of fungal diversity within the rice phyllosphere, surpassing conventional cultivation techniques [57]. This molecular insight augments our approach to rice crop management by advocating for a more comprehensive strategy. Although bacteria are widely recognized for their influence on rice health, endophytic fungi like *Trichoderma* and *Fusarium* have demonstrated significant biocontrol abilities, enhancing the plant’s resistance to pathogens such as *Rhizoctonia solani* and *Magnaporthe oryzae.* Table 2 presents a comprehensive overview of fungal diversity in the rice phyllosphere, detailing the relative abundances of various fungal groups and their ecological roles. This table highlights the predominance of *Ascomycota*, which constitutes a significant portion of the fungal community, alongside other important groups such as *Basidiomycota* and *Zygomycota*, emphasizing their contributions to plant health and nutrient cycling.

In addition to bacteria and fungi, viruses represent another important microbial component of the rice phyllosphere with significant impacts, as discussed below.

### 2.3. Virus Diversity in Rice Phyllosphere

The rice phyllosphere is a habitat not only for diverse bacterial and fungal communities but also for various viral populations. These viral communities can significantly influence rice plant health and productivity, acting either as pathogens or as beneficial agents that modulate microbial interactions [62,63]. The composition of viral populations in the phyllosphere includes plant viruses [63], bacteriophages [64], and mycoviruses [65].

Jiang et al. [66] conducted a study demonstrating that a combination of phages significantly reduced bacterial leaf blight caused by *Xanthomonas oryzae* pv. *oryzae* by 64.3%. The phage treatment not only decreased pathogen populations but also restored the balance of the phyllosphere microbiome, leading to an increase in microbial diversity. These findings suggest that phage therapy holds promise as an effective biocontrol strategy for managing bacterial diseases in rice. *Phyllosphere viruses*, particularly bacteriophages, may modulate bacterial populations, providing a natural defense against bacterial leaf blight.

In addition to bacteriophages, plant viruses in the rice phyllosphere also play a role in plant health. Viruses such as Rice yellow mottle virus and Rice stripe virus are known to cause significant yield losses in rice plants [67]. These viral infections can disrupt the microbial community in the phyllosphere by inducing stress responses in the host plant, which, in turn, can alter microbial dynamics. Studies have demonstrated that viral infections can shift the abundance of beneficial microbes, potentially weakening the plant’s defense mechanisms against other pathogens.

Table 3 provides a systematic overview of the virus species identified in the rice phyllosphere, categorized by their respective taxa. This table is essential for understanding the diversity and interactions among viruses in specific plant environments.

### 2.4. Infectious Cycle Stages of Rice-Associated Microorganism

The infectious cycle of rice-associated microorganisms within the rice phyllosphere encompasses a series of critical stages that govern microbial interactions with the rice plant, ultimately impacting plant health and productivity. The cycle commences with colonization, where microorganisms, including both beneficial bacteria and pathogenic fungi, adhere to the surfaces of rice leaves. Beneficial bacteria, such as *Methylobacterium*, establish themselves by utilizing nutrients from leaf exudates, fostering a symbiotic association. Conversely, pathogens like *M. oryzae* utilize specialized structures known as appressoria to penetrate the plant’s cuticle, initiating their invasive process [77].

Upon successful colonization, the cycle progresses to the infection stage, during which pathogens invade internal plant tissues and initiate reproduction. This invasion often involves the secretion of cell-wall-degrading enzymes or the production of toxins that interfere with normal cellular functions, manifesting in symptoms such as wilting and leaf spot formation [78,79].

As pathogens proliferate within rice plant tissues, they frequently elicit a host response. This defensive response prompts the rice plant to activate various defense mechanisms, including the production of reactive oxygen species (ROS) and defense-related proteins, which serve to inhibit pathogen spread [26]. Nonetheless, certain pathogens have evolved sophisticated mechanisms to bypass these defenses, allowing them to persist and continue their life cycle within the host [80].

The infectious cycle culminates in disease development, marked by the appearance of visible symptoms and potential yield loss [81]. During this phase, pathogens may produce resting spores or other survival structures, enabling persistence in the environment until conditions favor a new cycle of infection [82].

Understanding each stage of this infectious cycle is essential for devising effective disease management strategies in rice cultivation. Insights into the roles of beneficial microorganisms in enhancing plant resilience and the strategies pathogens employ to exploit host vulnerabilities can inform agricultural practices that foster robust microbial communities while minimizing disease incidence and severity.

## 3. Factors Influencing the Composition and Abundance of Rice Phyllosphere Microbes

**Figure 2:**
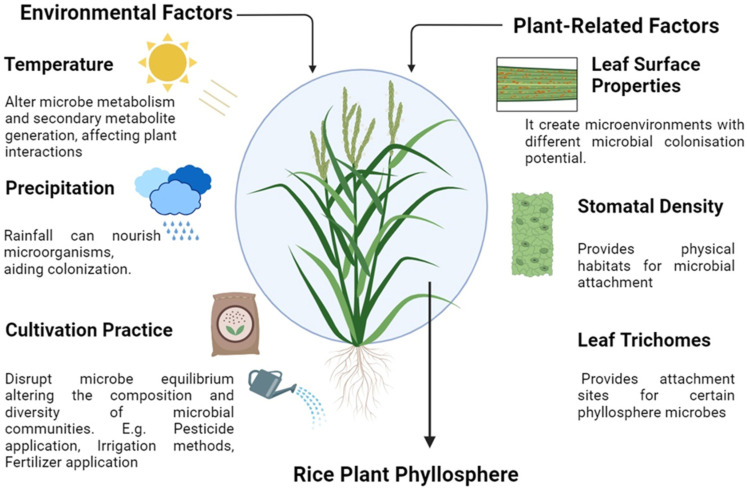
Illustration of factors influencing phyllosphere microbes in rice plants.

The composition and abundance of microorganisms in rice phyllosphere are influenced by various factors, including agricultural practices [83], environmental conditions [76], and the genetic diversity of rice genotypes [84], as illustrated in Figure 2. These elements play critical roles in shaping microbial community dynamics and their functional contributions to plant health.

### 3.1. Environmental Factors

Environmental factors such as temperature, humidity, light intensity, and soil conditions play a crucial role in shaping the microbial communities in the rice phyllosphere [14,20]. Research has consistently shown that humidity is a key factor in shaping microbial ecosystems. In particular, high-humidity conditions are conducive to the growth of harmful fungi, such as *Fusarium* and *Rhizoctonia*, which pose a threat to the health of rice plants [85,86]. In contrast, beneficial fungi, such as *Trichoderma*, showed robust resilience under moderate humidity, implying that the regulation of humidity can promote beneficial microbial dynamics [87]. Furthermore, [88] demonstrated that the rice phyllosphere in tropical zones, characterized by higher temperatures and humidity, exhibited greater bacterial diversity than that in temperate zones. This observation indicates that warmer and more humid environments may encourage a more diverse microbial community.

Research indicates that light intensity plays a critical role in shaping the phyllosphere, primarily by influencing photosynthesis and the subsequent production of plant exudates. These exudates provide nutrients for microbes, with higher light intensity enhancing microbial activity due to increased exudate production [14]. The phyllosphere-associated microbial communities exhibit adaptation to changing light conditions, causing temporal variations in microbial composition and function during the day [20]. Research by Kondo et al. [89] indicated that certain bacterial communities, including *Methylobacterium*, increased in abundance under optimal light conditions, contributing to nitrogen fixation and plant growth promotion. While a high light intensity can stimulate microbial activity through increased exudate availability, UV radiation can adversely affect microbial survival and population composition, given the vulnerability of many microbes to UV exposure [90]. UV-B radiation has been shown to promote the survival of UV-tolerant bacterial strains, thereby causing shifts in the community structure. As a result, this selective pressure can decrease microbial diversity, particularly in habitats subject to high levels of UV exposure [91]. These findings emphasize the role of light as a key factor in shaping microbial communities.

### 3.2. Plant Genotypes

One of the key factors shaping phyllosphere microbial communities is plant genotype [92]. Different rice varieties exhibit distinct microbial profiles, largely due to variations in leaf morphology, chemical composition, and physiological traits. These differences are thought to stem from the diverse chemical compositions of leaf exudates, which serve as nutrient sources for microorganisms [93]. Li et al. [94] demonstrated that a significant portion of the variation in phyllosphere microbial communities can be attributed to the host plant genotype. Their study highlighted how specific genotypes create more favorable conditions for certain microbial taxa, thereby exerting a decisive influence on microbial composition. For instance, wild rice genotypes tend to harbor more complex and stable microbial networks compared to cultivated varieties, indicating that genotype-driven selection of beneficial microbes plays a crucial role in shaping these communities [20].

The study named High-throughput sequencing analysis of microbial community diversity in response to indica and japonica bar-transgenic rice paddy soils, conducted by [95], found that microbial communities in the rhizosphere of *indica* and *japonica* rice were distinctly different, indicating the influence of rice genotype on microbial diversity. *Indica* varieties had higher microbial diversity compared to *japonica*, with a greater range of bacterial genera. Key differences in the dominant genera were noted: *Synechococcus* and *Dechloromonas* were more common in *japonica* soils, while *Chloronema*, *Flexibacter*, and *Blastocatella* were prevalent in *indica* soils. These findings highlight the significant role of plant genotype in shaping rhizosphere microbial composition.

Different rice genotypes exhibit distinct levels of bacterial populations and functional traits within the phyllosphere. For instance, the CR-1009 genotype supported the largest bacterial population, whereas P-44 and PS-5 demonstrated a higher presence of cyanobacteria [60]. Recent studies underscore the significance of genotype-specific responses in shaping the phyllosphere microbiome, indicating promising avenues for enhancing plant growth and improving disease resistance through targeted interventions tailored to host-specific attributes.

### 3.3. Agricultural Practices

Agricultural practices, including fertilization, irrigation, and crop management, can significantly influence the composition and abundance of rice phyllosphere microbes.

Fertilizer application can alter the composition of microbial communities across different phyla in the rice phyllosphere. This modulation extends to the functional genes involved in key processes such as carbon fixation and denitrification, emphasizing fertilization’s pivotal role in regulating biogeochemical cycles [83]. Thapa et al. [96] reported that fertilizer application significantly impacted the communities of *Bacteroidetes*, *Firmicutes*, *Planctomyces*, and *Proteobacteria*. Notably, the diversity of the *Firmicutes* phylum exhibited substantial variations influenced by both cultivation practices and fertilizer treatments.

Foliar fertilization does not influence the α-diversity of the phyllosphere bacterial community, but it can substantially modify the community structure by altering the chemical properties of the leaves [96]. Additionally, chemical fertilizers impact the composition of the phyllosphere microbiome [97]. According to Wu, Li, Chen, Jin, Wu, Li, Sun, Zhu, and Lin [83], nitrogen fertilization significantly alters fungal communities in the rice phyllosphere, affecting interactions between fungi and bacteria. Moderate nitrogen application (N210) encourages beneficial microbes and suppress pathogenic fungi, while high nitrogen levels (N330) promote the proliferation of pathogenic fungi. Thapa et al. [98] noted that the interaction between nitrogen levels and pathogens such as *M. oryzae* plays a crucial role in shaping the phyllosphere microbiome and modulating plant defense mechanisms. Optimal nitrogen application enhances photosynthetic pigments and stimulates defense enzyme activity, with distinct differences observed between resistant and susceptible rice cultivars [99].

Rice plants grown under different cultivation systems, such as the System of Rice Intensification (SRI) and conventional methods, display variations in physiological activity and microbial populations due to differing soil environments (aerobic vs. anaerobic) and variations in nutrient availability and leaf chemical properties [100,101]. Cyanobacterial populations in the phyllosphere are particularly influenced by cultivation methods like SRI [17]. The presence of cyanobacteria in the phyllosphere under SRI conditions contributes to enhanced plant growth by supplying essential nutrients and bolstering stress resistance, ultimately leading to improved plant health and higher yields [102,103]. Moreover, alternate wetting and drying (AWD) irrigation practices foster a more diverse microbial community in the phyllosphere by reducing water stress and increasing oxygen availability, which promotes the growth of beneficial microbes. Studies indicate that AWD enhances microbial richness and diversity, improving rice plant health and yield [93].

### 3.4. Climatic Factors

Geographical location plays a key role in determining the structure of microbial communities in the rice phyllosphere. Factors such as latitude, longitude, altitude, and local soil characteristics all contribute to the variations in microbial diversity [104]. Studies have demonstrated that rice plants cultivated in tropical regions typically exhibit greater microbial diversity compared to those grown in temperate zones [105]. Latitude-dependent mechanisms are known to regulate the diversity and biogeography of phyllosphere bacteria. In higher latitudes, host-related factors become increasingly significant in shaping bacterial communities, whereas at lower latitudes, abiotic factors and spatial proximity exert a greater influence [104].

This difference is driven by environmental factors, including temperature, humidity, and rainfall patterns. The agro-climatic zone also shapes microbial community assembly, as rice genotypes grown in similar zones tend to display converging phyllosphere microbiome profiles [15]. Conversely, rice plants cultivated in contrasting climatic conditions exhibit divergent microbial communities.

Ren et al. [106] reported that rising temperatures and the combined effect of elevated CO_2_ and temperature significantly alter the bacterial community structure in the rice phyllosphere, particularly in the upper leaves. Elevation gradients have a pronounced impact on the diversity and composition of phyllosphere microbial communities. In rice phyllospheres, bacterial diversity exhibits significant variation along these gradients, with elevation exerting a stronger influence on bacterial diversity than on fungal diversity [1]. Ueda et al. [107] also found that elevated ozone levels mildly impacted bacterial communities in both the phyllosphere and rhizoplane of rice, although the changes did not result in significant functional shifts.

### 3.5. Geographical Factors

The global landscape of rice cultivation exhibits notable disparities in plant genotypes and agricultural practices, particularly between Eastern and Western regions. These differences significantly influence the microbial dynamics within the rice phyllosphere, which, in turn, affects plant health and productivity [15].

In Eastern regions, such as Southeast Asia and South Asia, rice farming often emphasizes traditional agricultural practices that prioritize local varieties and organic methods [108]. Farmers in these areas typically cultivate indigenous rice genotypes that have co-evolved with their specific environmental conditions, fostering unique microbial associations within the phyllosphere [14,93]. For instance, traditional rice varieties in South Asia may support diverse microbial communities that enhance plant resilience against diseases and facilitate nutrient uptake [109]. The application of organic fertilizers and crop rotation in these regions further enriches microbial diversity, as these practices promote beneficial microorganisms capable of suppressing pathogens and improving soil health [110,111].

In contrast, Western regions like North America and East Asia tend to adopt more intensive agricultural practices characterized by the use of high-yielding rice varieties and synthetic fertilizers [112]. Such modern approaches can lead to a decline in microbial diversity within the phyllosphere due to a reliance on chemical inputs, which may disrupt natural microbial communities [113]. Research indicates that intensive farming practices can significantly alter the composition of the phyllosphere microbiome, often favoring specific bacterial taxa while diminishing others that are beneficial for plant health [114]. This shift can heighten the risk of disease outbreaks, as the absence of beneficial microbial competition may create an environment conducive to pathogen proliferation

## 4. Microbial Interactions Within the Rice Phyllosphere

The rice phyllosphere is a dynamic environment filled with diverse microorganisms that interact with one another and with rice plants, forming complex relationships that significantly affect plant health and productivity. These interactions are multifaceted and can be broadly classified into three categories: competitive, synergistic, and antagonistic [115], as summarized in Table 4.

### 4.1. Competitive Interactions

Competitive interactions within the rice phyllosphere primarily arise as microorganisms compete for limited resources such as nutrients, space, and light [116]. For example, bacterial populations often compete for specific micro-niches on the leaf surface [117]. Research has shown that certain bacterial strains dominate these niches due to their superior growth rates or ability to efficiently utilize available resources. According to Schlechter, Kear, Bernach, Remus, and NP [117], metabolic resource overlap significantly impacts competition among phyllosphere bacteria. They observed that the growth of *Pantoea eucalypti* 299R (Pe299R) is affected by other bacterial colonizers, with resource overlap playing a crucial role in competitive dynamics. *Pseudomonas syringae*, a common phyllosphere bacterium, can outcompete other species by producing compounds that enhance its nutrient uptake or inhibit competitor growth [118]. Meena et al. [119] found that some beneficial bacteria isolated from the rice phyllosphere possess plant-growth-promoting traits, enabling them to outcompete pathogenic microbes for resources.

Additionally, competitive interactions can drive shifts in community composition. The presence of dominant species may suppress microbial diversity, leading to monocultures that are less resilient to environmental stresses [93]. This is particularly significant in rice cultivation, where a diverse microbial community is linked to improved plant health and disease resistance.

### 4.2. Synergistic Interactions

In contrast to competition, synergistic interactions occur when microorganisms collaborate, enhancing each other’s growth and survival. These interactions are essential in the rice phyllosphere for nutrient acquisition, stress resistance, and overall plant health [120]. Bacteria and fungi often form symbiotic relationships that facilitate resource sharing. For instance, *Lactobacillus* spp. and *Aspergillus* spp. in the rice phyllosphere play a key role in disease resistance by inducing metabolic defenses against pathogens such as *Ustilaginoidea virens*, the causative agent of false-smut disease [21]. Bandyopadhyay et al. [121] reported that the combination of *Piriformospora indica*, an endophytic fungus, and *Azotobacter chroococcum*, a nitrogen-fixing bacterium, significantly enhances nutrient uptake and plant growth in rice by upregulating proteins involved in nitrogen and phosphorus metabolism. Similarly, [122] demonstrated that the interaction between *Serendipita indica* and *Zhihengliuella* sp. ISTPL4 promotes rice growth by increasing spore germination and improving biochemical parameters, such as chlorophyll and flavonoid content.

Sobanbabu et al. [123] reported that *Pseudomonas aeruginosa* and *Bacillus subtilis* effectively inhibit the growth of foliar pathogens such as *Bipolaris oryzae* and *Sarocladium oryzae*, thereby reducing disease incidence when applied to rice plants.

Synergistic interactions in the rice phyllosphere present numerous potential benefits, though they are often complex and context-dependent. For example, the efficacy of biocontrol agents may fluctuate based on the specific pathogen they target, while certain microbial communities might foster antagonistic interactions instead of the expected synergy [124]. A deeper understanding of these dynamics is essential to optimize the use of synergistic interactions in agricultural practices.

### 4.3. Antagonistic Interaction

Antagonistic interactions, where one organism harms another, are prevalent in the rice phyllosphere and occur through mechanisms like antimicrobial compound production or direct pathogen inhibition [123,125]. For example, certain bacteria produce secondary metabolites toxic to pathogenic fungi, protecting rice plants from diseases like leaf blight and bacterial blight [126]. Biocontrol agents play a crucial role in managing plant pathogens. Bacteria such as *Bacillus subtilis* are extensively studied for their ability to suppress fungal pathogens via competitive exclusion and the production of lytic enzymes [123]. Similarly, *Trichoderma* spp. secrete lytic enzymes that degrade the cell walls of pathogenic fungi, providing biocontrol against fungal infections. Direct antagonistic effects against *Rhizoctonia solani* are observed in *Trichoderma harzianum* and *Pseudomonas fluorescens*, which employ mechanisms like hyphal coiling, mycoparasitism, and the production of siderophores and antibiotics [125]. Endophytic bacterial consortia have been shown to suppress the growth of *Rhizoctonia solani* and *Xanthomonas oryzae* pv. *oryzae* via antibiosis, inducing morphological changes in these pathogens [127]. Additionally, *Streptomyces hygroscopicus* produces antimicrobial compounds that effectively inhibit both bacterial and fungal pathogens, demonstrating its potential for formulating starter culture powders aimed at pathogen suppression [128]. These agents not only reduce disease incidence but also support healthier plant growth by maintaining a balanced microbial community on leaf surfaces. In addition to direct suppression, antagonistic interactions influence plant immune responses by modulating the salicylic acid and jasmonic acid pathways, both crucial for resistance to diverse pathogens [129]. Sobanbabu, Oviya, Meena, Vijayasamundeeswari, Shanmugaiah, and Ramamoorthy [123] highlighted that resident phyllosphere bacteria, fungi, and actinomycetes exhibit biocontrol activities that suppress plant pathogens. Furthermore, the effectiveness of these antagonistic interactions depends on environmental factors such as humidity, temperature, and the overall health of the rice plant [130,131]. Favorable conditions allow beneficial microorganisms to thrive and outcompete pathogens, whereas stress conditions can disrupt microbial dynamics, potentially enabling pathogen establishment. Table 4 illustrates key microbial interactions within the rice phyllosphere that significantly affect plant health and productivity.

**Table 4:** Key microbial interactions in the phyllosphere of rice plants and their effects on plant health.

Microbes Interaction	Interacting Microbes	Effect on Rice Plant	References
Competitive interaction	*Pseudomonas* spp. *Various saprophytic fungi*	Produces antimicrobial compounds to inhibit competitors. Compete for nutrients and space, limiting pathogen growth.	[132,133]
Antagonistic interaction	*Trichoderma* spp. vs. fungi	Biocontrol of fungal pathogens.	[134,135]
	*Pseudomonas* spp. vs. *Xanthomonas* spp.	It suppresses bacterial and fungal pathogens.	[136]
Synergistic interaction	Mycorrhizal fungi and plants	It enhances nutrient uptake and plant growth	[137,138]
	Algae and bacteria	It induces drought tolerance in rice and improves photosynthesis efficiency.	[139,140]
	Actinobacteria (*Streptomyces* spp.) and plants	It enhances nutrient cycling and nutrient availability.	[141,142]
	*Rhizobia* spp. and rice plant	It encourages the proliferation of rice plants.	[143]
	*Methanobacterium* spp. and Methanotrophic bacteria	It impacts methane release and the cycle of nutrients.	[144,145]

## 5. Beneficial Microbes and Plant-Growth-Promoting Bacteria

Plant-growth-promoting bacteria (PGPB) in the rice phyllosphere play a pivotal role in enhancing rice plant growth by supporting various physiological processes. These beneficial microbes contribute to several plant-growth-promoting (PGP) mechanisms, including improving nutrient availability, boosting stress tolerance, and suppressing plant pathogens [37]. Unlike chemical fertilizers and pesticides, PGPB offer a sustainable way to protect rice plants from bacterial diseases and enhance overall plant health.

PGPB directly stimulate plant growth through multiple mechanisms. For instance, bacteria such as *Methylobacterium* are known for producing phytohormones like indole-3-acetic acid (IAA), which enhances nutrient absorption [146,147]. They also produce other phytohormones, such as gibberellins and cytokinins, which stimulate rice plant growth and development [37]. These bacteria further aid nutrient acquisition by solubilizing phosphorus and producing siderophores that chelate iron, making these essential nutrients more accessible to the rice plant [148]. *Bacillus* species, commonly found in the rice phyllosphere, are particularly effective in enhancing nutrient uptake, especially under stress conditions [149].

In addition to promoting growth, beneficial microbes in the rice phyllosphere protect plants from phytopathogens by producing antimicrobial compounds like antibiotics and hydrogen cyanide, which suppress the growth of harmful bacteria and fungi [123]. Additionally, *Bacillus* sp. *KK281* produces lipopeptides with significant antimicrobial activity against pathogens like *Curvularia lunata* and *Xanthomonas oryzae* [150]. PGPB also compete with pathogens for nutrients and space, limiting their proliferation [151]. Furthermore, these microbes can induce systemic resistance in rice plants, priming their defense mechanisms against potential threats [152,153]. Species like *Pantoea* and *Trichoderma* have been shown to work synergistically, promoting rice growth while simultaneously reducing foliar diseases [154,155].

The rice phyllosphere microbiome also contributes to plant tolerance to abiotic stresses such as drought, salinity, and heavy metal toxicity. PGPB produce exopolysaccharides and osmoprotectants that help rice plants maintain cellular turgor, protecting them from osmotic stress [156]. Additionally, these bacteria secrete enzymes such as ACC deaminase, which lowers stress-induced ethylene levels in plants, thereby promoting growth under adverse conditions [157].

The application of phyllosphere microbes as biofertilizers for rice cultivation has garnered significant attention due to their potential to improve plant health and productivity. Research highlights the effectiveness of bacterial strains like *P. aeruginosa* and *B. subtilis* in managing foliar diseases such as brown spot and sheath rot while enhancing plant growth and disease resistance [123]. Commercial products such as Mikrobat, a biofertilizer containing beneficial microbes like Azotobacter and Pseudomonas, have demonstrated significant benefits, including improved soil health, increased seed vigor, and enhanced rice yields, reporting 12.74 tonnes per hectare compared to 9.42 tonnes in untreated controls [158]. Similarly, the commercial biofertilizer BioGro, formulated with four bacterial strains, has consistently shown positive effects on rice grain yield and other agronomic parameters [159]. Additionally, microbial biostimulants in India, often employing bacteria like *P. fluorescens* and *Bacillus* spp., serve multifunctional roles in supporting agricultural productivity [160]. Despite these advancements, challenges such as ensuring product quality control and addressing gaps in field efficacy research remain critical to achieving the broader adoption of these technologies.

## 6. Microbial Contribution to Nutrient Cycling and Nitrogen Fixation

Diverse microbial communities, including key genera like *Methylobacterium* and *Sphingomonas*, inhabit the rice phyllosphere and play critical roles in nutrient cycling and plant growth promotion [14]. These microbes exert significant influence on major nutrient cycles through intricate biochemical processes. Long-read metagenomic sequencing has uncovered novel bacterial species in the rice phyllosphere, revealing genetic diversity and potential new functional genes related to nutrient cycling [12]. Microbial interactions within the phyllosphere enhance nutrient cycling through processes such as transport, stress responses, and one-carbon conversion activities [161]. Notably, one-carbon conversion processes, including methanogenesis, methanotrophy, and methanol-based methylotrophy, are crucial for maintaining nutrient balance [162]. C1-microorganisms, particularly methanol-utilizing *Methylobacterium* spp., utilize C1-compounds emitted by plants, enhancing CO_2_ fixation and contributing to both plant growth and the global carbon cycle, thereby improving rice crop yields [163].

Atmospheric nitrogen fixation is another vital process in the rice phyllosphere, with nitrogen-fixing bacteria supplying essential nitrogen to plants and reducing reliance on chemical fertilizers [89,164]. Though less abundant than in the rhizosphere, nitrogen-fixing microbes in the phyllosphere, such as *Exiguobacterium*, *Bacillus*, and *Bradyrhizobium*, contribute to plant growth by promoting nitrogen fixation, stress resistance, and nutrient uptake, leading to enhanced rice grain yields when combined with appropriate fertilization [83,165]. These bacteria are essential for maintaining soil fertility and promoting sustainable rice cultivation. A consortia of phyllosphere and rhizosphere microbes also fix nitrogen, solubilize phosphorus, and secrete growth hormones, collectively stimulating rice plant development and improving growth and yield compared to conventional fertilization methods.

## 7. Biocontrol Agents Against Rice Pathogens

Plant-growth-promoting bacteria (PGPB) play a crucial role in enhancing plant growth, either directly through various physiological activities or indirectly via biocontrol mechanisms. Direct stimulation includes nitrogen fixation [166], phosphate solubilization [167], iron sequestration [147], phytohormone production [168], and the regulation of plant ethylene levels [169]. In contrast, biocontrol mechanisms involve the production of antibiotics, fungal cell wall-degrading enzymes, siderophore activity, and the induction of systemic resistance within plants [131,170].

Several studies have explored the potential of rice phyllosphere microbes as biocontrol agents against rice pathogens. Wang et al. [171] identified *Burkholderia pyrrocinia* S17-377 as a promising biocontrol agent against rice sheath blight caused by *Rhizoctonia solani*. The strain exhibited significant antifungal activity, with a 64.81% efficacy in pot experiments and 55.78% in field trials. In addition to its high inhibition rates against *R. solani*, S17-377 was shown to induce the expression of defense-related genes in rice and produce antimicrobial compounds, such as proteases and chitinases. These findings suggest that *B. pyrrocinia* S17-377 can effectively manage rice pathogens, demonstrating its potential for sustainable agricultural practices in rice production.

Do et al. [172] identified *Bacillus velezensis* BTR11 as a promising biocontrol agent against bacterial leaf blight (BLB) caused by *Xanthomonas oryzae* pv. *oryzae*. This endophytic bacterium exhibits significant antagonistic activity, with protective effects of up to 85% when combined with soil amendments and foliar sprays. Additionally, *B. velezensis* BTR11 promotes plant growth by releasing phytohormones, such as indole acetic acid, and mineralizing nutrients, leading to an approximate 12% increase in rice yield compared to untreated controls. These findings underscore the potential of utilizing endophytic bacteria in the rice phyllosphere for effective disease management and enhanced crop productivity

Furthermore, Sowmya et al. [173] demonstrated the effectiveness of native bioagents, including *Trichoderma asperellum*, *Bacillus cabrialesii*, and *Pseudomonas* species, in controlling rice pathogens such as *Sclerotium hydrophilum* and *Ustilaginoidea virens*. These bioagents not only reduced disease severity but also promoted plant growth and activated defense enzymes in rice. Their study highlighted that these microorganisms release various phytohormones, including indole acetic acid (IAA), gibberellic acid (GA), salicylic acid (SA), abscisic acid (ABA), and zeatin. These phytohormones help induce systemic resistance, offering a natural defense mechanism against stem rot and false smut, thereby demonstrating a sustainable approach to disease management in rice cultivation.

Table 5 summarizes the potential of various phyllosphere microbes as biocontrol agents against rice pathogens, detailing their specific mechanisms of action.

## 8. Chemical Defense Mechanisms of the Rice Phyllosphere Against Pathogens

Rice employs a critical strategy in disease defense by producing secondary metabolites—bioactive compounds that strengthen plant defense mechanisms. These metabolites not only directly inhibit pathogens but also modify the microbial community in the phyllosphere, promoting beneficial interactions and suppressing harmful ones.

Rice plants synthesize various secondary metabolites, which can be categorized into several groups based on their defensive roles. Phenolic compounds, such as flavonoids and phenolic acids, possess strong antioxidant properties and disrupt pathogen cellular functions [183]. For example, flavonoids are known to inhibit the growth of *Magnaporthe oryzae*, the fungus responsible for rice blast disease [184]. Terpenoids, another important group, exhibit antimicrobial activity, deterring herbivores and inhibiting fungal pathogens by disrupting cellular processes [185]. Additionally, terpenoids participate in signaling pathways that activate plant defenses [186]. Alkaloids, nitrogen-containing compounds, exert toxic effects on herbivores and pathogens, further enhancing the plant’s defense. Saponins, meanwhile, destabilize microbial membranes and are effective against a wide range of pathogens [187].

These secondary metabolites act through several mechanisms. Direct toxicity towards pathogens, such as the inhibition of fungal and bacterial growth by phenolic compounds, is a primary defense function [188]. Furthermore, secondary metabolites can activate systemic acquired resistance (SAR) in rice, stimulating the production of signaling molecules like salicylic acid, which boosts the expression of pathogenesis-related (PR) genes and primes the plant for future infections [189].

In addition to pathogen suppression, certain secondary metabolites selectively promote the growth of beneficial microbes in the phyllosphere. For instance, some phenolic acids support the growth of beneficial bacteria such as *Pseudomonas* spp., which provide biocontrol by producing antibiotics or outcompeting pathogens for resources [190]. These metabolites may also exert allelopathic effects, reducing competition and pathogen incidence while fostering a healthier phyllosphere microbiome [191].

The rice phyllosphere microbiome plays a significant role in plant defense by interacting with secondary metabolites to enhance plant health [192]. Beneficial microbes, including some endophytic bacteria, produce compounds that mimic plant hormones, promoting plant growth and improving stress resistance [193].

**Figure 3:**
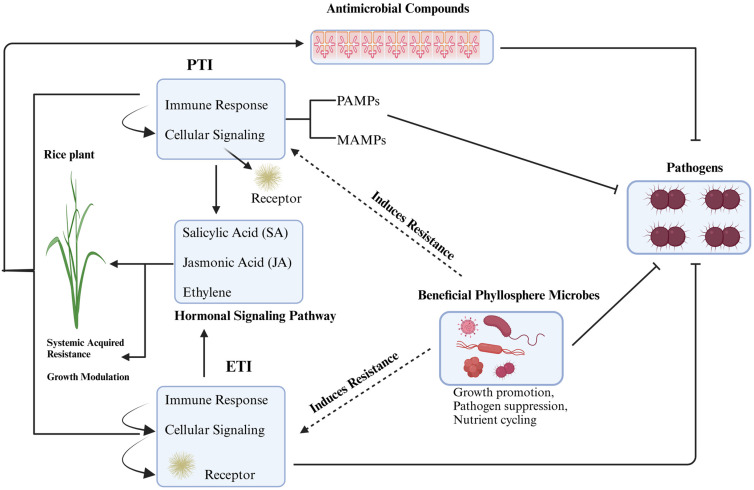
Diagram illustrating the plant immunity system in rice leaves, highlighting the PTI (PAMP-triggered immunity) and ETI (effector-triggered immunity) pathways and the role of antimicrobial compounds in defending against phyllosphere pathogens.

As shown in Figure 3, the immune system in rice leaves utilizes a variety of defense mechanisms to identify and counteract microbial threats. This system includes both pattern-triggered immunity (PTI) and effector-triggered immunity (ETI) pathways, which are essential for recognizing pathogenic invaders. In addition, rice plants produce antimicrobial compounds that serve as a crucial line of defense against phyllosphere pathogens, collectively reinforcing their resilience to microbial infections.

## 9. Synergies and Dynamics Between the Rhizosphere and Phyllosphere in Rice

The interplay between the rhizosphere and phyllosphere in rice plants represents a dynamic network of microbial communities that is central to plant health, growth, and productivity [194]. The rhizosphere, or the root-surrounding soil zone, contains abundant microorganisms that enhance nutrient absorption, stimulate growth, and guard against pathogens. Certain rhizosphere bacteria, such as those influenced by the *OsCIPK2* gene, enhance nitrogen uptake in rice by restructuring the root microbial community and promoting nitrogen-fixing bacteria under low-nitrogen conditions [195]. On the other hand, the phyllosphere, which includes the aerial portions of the plant, supports a diverse microbial ecosystem that impacts photosynthesis, disease resistance, and overall plant health [14]. Although spatially distinct, these zones are interlinked primarily through microbial interactions that facilitate nutrient cycling, stress resilience, and symbiotic relationships that support rice cultivation [196].

One important symbiotic relationship is with plant-growth-promoting rhizobacteria (PGPR), which colonize rice roots in the rhizosphere, enhance nutrient availability, and protect the plant from harmful pathogens [197]. These bacteria, such as *Pseudomonas aurantiaca* and *Pseudomonas chlororaphis*, are effective in mobilizing essential nutrients, such as zinc and potassium, thereby improving plant biomass and yield. Their ability to produce antimicrobial metabolites further contributes to the overall health and productivity of rice grown in nutrient-deficient soils [198]. Furthermore, studies have shown that inoculation with plant-growth-promoting bacteria (PGPB), such as *Bacillus velezensis*, can enhance microbial communities in both compartments, leading to improved plant growth [199]. This relationship contributes significantly to root-zone microbial activity and is essential for optimal rhizosphere growth. Additionally, microbial networks, both within and between these zones, contribute to nutrient cycling [200], reinforcing soil health [201] and making these ecosystems a cornerstone for sustainable rice farming [100]. Healthy soil environments encourage these beneficial interactions, resulting in improved rice productivity [165].

Although cooperative interactions between microbial communities in the rhizosphere and phyllosphere are beneficial, competitive dynamics also occur. Microbes may compete for resources like nutrients and space, leading to antagonistic relationships that can hinder plant growth and health [202,203]. Understanding these competitive processes is crucial for effectively managing microbial communities to support sustainable agriculture. Furthermore, endophytes, microorganisms residing within plant tissues, play a pivotal role in bridging the rhizosphere and phyllosphere [204]. They support nutrient uptake and stress tolerance, contributing to plant resilience under various environmental conditions [77].

These complex microbial interactions not only facilitate nutrient acquisition but also offer natural disease suppression mechanisms. Leveraging these mechanisms may lead to environmentally friendly disease management practices in rice cultivation [88]. Ultimately, understanding and managing the synergies between the rhizosphere and phyllosphere is key to optimizing rice productivity, fostering plant health, and advancing sustainable farming methods.

## 10. Implications for Crop Productivity and Health

The phyllosphere has increasingly attracted the attention of researchers because of its substantial influence on agricultural productivity [120,205]. Maintaining a well-balanced phyllosphere microbiome is vital for optimizing crop yields because it promotes symbiotic relationships that support the proliferation of beneficial microbes, which often suppress harmful diseases [13]. These microbes play a dual role by not only combating pathogens but also forming mutualistic associations with plants that improve nutrient uptake and bolster plant defenses against various diseases [13,206]. Consequently, fostering a harmonious phyllosphere is fundamental to ensuring crop health and resilience.

At the molecular level, advanced techniques, such as metagenomics and transcriptomics, have provided deeper insights into the dynamics of the phyllosphere microbiome. These methods help unravel the genetic interactions underlying plant–microbe relationships, revealing how beneficial associations can be enhanced while minimizing the impact of harmful ones [207]. Such molecular insights hold the potential to facilitate the development of improved crop varieties and innovative biocontrol agents [208]. Given the growing concerns over chemical interventions in agriculture, these molecular approaches offer a sustainable alternative, rooted in natural processes.

In the context of sustainable agriculture, microbial inoculants have emerged as transformative tools. These treatments, enriched with beneficial microorganisms, enhance the plant’s native microbial community and offer numerous benefits, including improved soil fertility and strengthened plant defenses [209]. The environmental and economic advantages of microbial inoculants are particularly notable, as nitrogen-fixing bacteria can reduce the need for synthetic fertilizers, thus mitigating both financial and environmental costs [210]. Moreover, specific microbial treatments can “prime” plants for defense, increasing their resilience to future pathogenic challenges [211]. In light of the global challenges of food security and sustainability, microbial inoculants grounded in natural strategies present a promising path forward.

## 11. Future Perspectives and Challenges

The study of rice phyllosphere microbiomes has made significant strides in recent years, offering unprecedented insights into the complex interactions between plants and their microbial communities. The field presents both exciting opportunities and formidable challenges.

One of the primary challenges in studying the rice phyllosphere microbiome is the difficulty in obtaining representative samples without the contamination or disruption of microbial communities. The delicate nature of the phyllosphere environment requires careful sampling techniques to ensure an accurate representation of the microbial populations. Additionally, researchers face the ongoing debate between culture-dependent and culture-independent methods. While culture-independent strategies, such as 16S rRNA gene amplicon sequencing, have provided unprecedented insights into the phyllosphere microbial composition, they are not without limitations. These methods can introduce biases in PCR amplification and may not distinguish between live and dead microorganisms, potentially skewing our understanding of the active microbial community. Recent studies have highlighted additional challenges, such as the need to better understand the spatial and temporal dynamics of microbial populations, their interactions with the host plant, and their genetic adaptations. Moreover, integrating inter-kingdom microbial interactions, improving taxonomic and functional resolution of sequencing data, and considering spatial and temporal factors are crucial for advancing research in this area.

Analytical challenges persist in moving beyond taxonomic profiling to understanding the functional roles of microbes in the phyllosphere. The complex interactions between microorganisms and their host plant, as well as among different microbial species, make it difficult to elucidate specific functional contributions. Furthermore, the high environmental variability in the phyllosphere poses challenges for the reproducibility and generalizability of studies. Factors such as climate, agricultural practices, and plant genotype significantly influence microbial communities, making it crucial to consider these variables when interpreting results and designing experiments. Metabarcoding and culturomic approaches have revealed diverse bacterial communities in the rice phyllosphere, with potential for suppressing rice blast disease, while metaproteogenomic analysis has shed light on microbial physiology, such as the prevalence of methylotrophy in the phyllosphere and methanogenesis in the rhizosphere. These findings demonstrate the need for multifaceted approaches to overcome the current limitations in microbiome research.

Looking towards the future, there is a pressing need for standardized protocols in phyllosphere microbiome research to enhance comparability between studies. Emerging technologies like single-cell genomics and metabolomics hold promise in addressing current limitations by providing more detailed insights into individual microbial functions and interactions. However, translating laboratory findings to field applications remains a significant challenge, particularly in the context of biocontrol agents like *Bacillus subtilis*. Future research should prioritize comparative field trials across diverse ecosystems to better understand the factors influencing the effectiveness of these agents.

The phyllosphere serves as a crucial indicator of the health and resilience of agricultural ecosystems, particularly in the context of rapid global climate change. Understanding phyllosphere dynamics is vital as climate conditions become more unstable. Microbial populations in the phyllosphere can buffer against drought, heat, and salinity, thereby increasing plant resilience. They also influence plant responses to changes in CO_2_ levels and precipitation. Research on the phyllosphere in the context of climate change can lead to the development of future-proof crops, ensuring food security in unpredictable environments.

Another significant challenge lies in developing efficient delivery systems for beneficial microbes. Ensuring that introduced microbes can establish and persist in the phyllosphere under field conditions requires innovative approaches. This includes formulating microbial inoculants that are stable, effective, and easy to apply on a large scale. Moreover, adapting irrigation techniques, such as alternate wetting and drying, has been shown to foster beneficial microbial communities in the rice phyllosphere. Furthermore, there is a need for regulatory frameworks that support the use of microbial products in agriculture while ensuring their safety for humans and the environment. Public acceptance and awareness of microbial-based technologies also play a crucial role in their adoption. Economic viability, logistical constraints, and ecological implications must be considered when scaling-up technologies such as microbial inoculants or molecular insights. To maximize the promise of phyllosphere research, a holistic strategy that incorporates cutting-edge science and practical realities is essential.

## 12. Conclusions

The complex interactions between rice plants and bacteria inhabiting the phyllosphere present an opportunity to improve crop output, resilience, and overall plant health. The present study elucidated the diverse functions of these microorganisms, underscoring their importance in nutrient cycling, disease control, and facilitation of plant growth. Mutualistic interactions emphasize the potential for utilizing phyllosphere bacteria to achieve sustainable rice agriculture. Given the growing complexities associated with climate change, insect resistance, and global food security, it is imperative to understand and harness the potential for microbial collaboration. As research in this field continues to advance, it is becoming increasingly crucial for scientists and agricultural practitioners to incorporate these findings into contemporary agricultural methodologies. By using this approach, we can guarantee the long-term viability of rice farming and facilitate the development of novel techniques that can be applied to other agricultural commodities, thereby enhancing global food stability.

## Figures and Tables

**Table 1:** Predominant bacterial groups in the rice plant phyllosphere and their functional roles.

Dominant Taxa	Roles	Mechanism of Action	References
*Bacillus*	It boosts rice growth and protects against drought, making the plant more drought-tolerant.	It promotes plant growth by producing compounds such as siderophore, phytase, and indole acetic acid (IAA).	[28,29]
*Erwinia*	It facilitates xylem colonization and movement.	It establishes itself at infection sites and effectively outcompetes pathogens, ensuring their exclusion.	[30]
*Exiguobacterium*	It promotes plant growth, including fixing nitrogen. It can help reduce drought stress in rice plants.	It helps reduce drought stress in rice by producing compounds that support growth and relieve stress.	[31,32]
*Sphingomonas*	It exerts a positive influence on rice growth and the ultimate grain yield.	It fights melanose pathogens in the phyllosphere by competing for iron and producing antimicrobial substances, such as antibiotics and cell-wall-digesting enzymes.	[33]
*Methylobacterium*	It helps plants by providing nutrients, regulating hormones, and protecting against diseases, making it an effective growth-promoting bacterium.	This is achieved through the synthesis of phytohormones like auxin and cytokinins, coupled with its inherent antibacterial activities.	[34,35]
*Microbacterium*	It plays a pivotal role in enhancing rice plant growth and resistance to abiotic stresses within the phyllosphere.	It acts as a beneficial microbe in rice leaves, helping dissolve nutrients and produce enzymes and plant hormones.	[36,37]
*Pantoea*	It plays a significant role in augmenting the drought resilience of rice seedlings.	This is attributed to its capacity for phosphate solubilization, synthesis of siderophores and exopolysaccharides, and the production of antibiotics.	[38,39]
*Pseudomonas*	They amplify plant growth, shield against pathogenic entities, and facilitate optimal nutrient absorption.	They achieve this by producing potent antimicrobials, including antibiotics, and secreting cell-wall-degrading enzymes like endo-β-1,3-glucanase, chitinase, and cellulase.	[40,41]
*Streptomyces*	They are crucial in preventing the spread of the fungus that causes rice blast disease. They help rice plants resist iron deficiency by affecting processes like iron chelation, solubilization, reduction, and transport.	This is achieved through mechanisms that compromise the cell wall and membrane integrity and perturb mitochondrial functions in the rice blast pathogen.	[42,43]

**Table 2:** Fungal diversity in the rice phyllosphere: relative abundance and ecological roles.

Fungal Group	Relative Abundance	Description	References
*Ascomycota*	55–74%	Predominant in rice phyllosphere, with higher presence in organically managed fields.	[1,58]
*Basidiomycota*	2–10%	Generally present in lower abundance; varies with elevation and environmental factors.	[1]
*Aspergillus* spp.	Variable	Dominates asymptomatic rice phyllosphere; inhibits rice pathogens like *Magnaporthe oryzae*.	[59]
*Xylaria*, *Gibberella*, *Penicillium*	Variable	Common in rice at various elevations; presence correlates with soluble protein levels in leaves.	[60]
*Zygomycota*	5–15%	They are often involved in the decomposition of organic matter and nutrient cycling.	[61]

**Table 3:** Virus species and their corresponding taxa that exist in the rice phyllosphere.

Virus Species	Taxonomic Family	Mode of Transmission	Effects on Rice Plant	References
Rice Stripe Virus (RSV)	*Tenuivirus*	Small brown planthoppers	Yellow stripes on leaves, reduced growth	[68,69]
Rice Tungro Virus (RTBV)	*Caulimoviridae*	Green leafhoppers	Stunting, reduced tillering, yield loss	[70,71]
Rice Grassy Stunt Virus (RGSV)	*Tenuivirus*	Planthoppers	Stunted growth, reduced tillering	[63,72]
Rice Yellow Mottle Virus (RYMV)	*Sobemovirus*	White-backed planthopper	Yellowing, reduced photosynthesis, yield loss	[67,73]
Rice Black-Streaked Dwarf Virus (RBSDV)	*Reoviridae*	Small brown planthoppers	Stunted growth, dark streaks on leaves	[74,75]
Rice Necrosis Mosaic Virus (RNMV)	*Sobemovirus*	Brown planthoppers	Necrotic lesions and yellowing on rice leaves.	[76]

**Table 5:** Potential of phyllosphere microbes as biocontrol agents against rice pathogens and their mechanisms of action.

Pathogen	Phyllosphere Microbe	Mechanism of Biocontrol	References
Sheath Blight	*Trichoderma* spp.	It establishes mycoparasitic interactions with fungal pathogens. It secretes lytic enzymes responsible for the breakdown of fungal cell walls.	[174,175]
	*Streptomyces* spp.	It curtails the proliferation of pathogens via antibiosis mechanisms. It bolsters the defensive reactions of rice plants.	[43,176]
Bacterial Blight	*Bacillus* spp.	It elicits systemic resistance within rice plants. Additionally, it secretes antibiotics that act as deterrents to pathogen expansion.	[172,177]
	*Pseudomonas* spp.	It vies for both spatial and nutritive resources against pathogens. Concurrently, it synthesizes antimicrobial agents that counteract pathogenic organisms.	[178]
Rice Blast	*Burkholderia* spp.	It secretes antifungal metabolites, specifically targeting blast fungus. Additionally, it triggers systemic resistance within rice plants.	[26,171]
	*Ampelomyces quisqualis (fungus)*	It parasitizes the spores of rice blast, thwarting their germination process. Furthermore, it mitigates the intensity of the disease.	[179]
Rice Tungro Virus	*Metarhizium anisopliae*	It synthesizes antiviral agents that curtail virus replication and amplify the immune response of rice plants.	[180]
Brown Spot	*Methylobacterium* spp.	It emits volatile organic compounds with the capability to deter pathogen proliferation. Additionally, it bolsters the defense mechanisms of plants.	[181]
	Non-pathogenic *Xanthomonads*	It holds a competitive edge over pathogenic strains, vying for nutritive resources and spatial occupancy, leading to their inhibition.	[182]

## Data Availability

No new data were created or analyzed in this study.
